# Problems with etiologic diagnosis of community-acquired pneumonia using plasma microbial cell-free DNA sequencing

**DOI:** 10.1017/ash.2023.475

**Published:** 2023-11-09

**Authors:** Daniel M. Musher

**Affiliations:** ^1^ Michael E. DeBakey Veterans Affairs Medical Center, Houston, TX, USA; ^2^ Baylor College of Medicine, Houston, TX, USA

In a retrospective study, my esteemed colleagues^1^ reported results of plasma next-generation sequencing (“Karius test” [KT]) in 167 patients in a large tertiary-care hospital. They hypothesized that high sensitivity of KT would encourage physicians to reduce antibiotic use and, consistent with their hypothesis, they found that glycopeptide use was reduced. However, the specificity of KT, which is essential to antibiotic stewardship, was not addressed. I am concerned that dependence on the results of KT will lead to excessive antibiotic use, thereby opposing antibiotic stewardship.

In their study, KT identified potential pathogen(s) in 118 of 167 patients (70.7%), a single bacterium in 50 (42.4%), and 2 to 10 organisms (57.6%) in the remainder. Their endpoint was discontinuation of antibiotics based on KT results, which were available, on average, 3 d after specimen collection but, importantly, an unstated number of days after antibiotics had been begun.

The principal question, however, is not whether antibiotics were discontinued but whether KT yielded a correct microbiologic diagnosis, a concern that is reinforced by KT’s finding of polymicrobial infection in many patients and discordance between KT and blood culture results in 4 of 11 cases with positive blood cultures.

Using previously described methods,^2^ we addressed the accuracy of KT in a small number of meticulously studied patients hospitalized (pre-COVID) for community-acquired pneumonia (CAP), in whom a microbiologic diagnosis was clearly established. We compared KT to quantitative bacteriology (QB) of sputum in 11 patients: (1) who provided purulent sputum at the time of or shortly after admission, (2) whose Gram-stained sputum showed >20 white blood cells (WBC) per epithelial cell, (3) whose blood cultures were negative, and (4) who had received minimal or no antibiotic therapy. Plasma obtained at admission was assayed by KT. A nasopharyngeal swab was studied for viral respiratory pathogens using bioMérieux BioFire FilmArray Respiratory Panel.

The median time of antibiotic therapy before sputum was obtained was 0 h (Table 1; range 0–5 h). Gram stain readings were consistent with the results of QB in every case. In three patients (#1–3), QB of sputum and KT yielded fully concordant results. In two patients (#4,5), KT was negative, but QB yielded high cfu/ml of typical respiratory pathogen(s). In patient #6, KT identified *Streptococcus pneumoniae* that was not detected by QB; pneumococcus may have been missed by QB because of the presence of other streptococci in high numbers. In patient #7, QB was negative (<10^3^ cfu/ml), routine sputum culture yielded scant growth, and PCR of a nasopharyngeal swab was positive for rhinovirus. The final diagnosis was rhinovirus pneumonia without bacterial coinfection, but KT was positive for *S. pneumoniae* and *Moraxella catarrhalis*, which we regard as falsely positive. In the remaining four patients (#8–11), results were discordant, with KT identifying organisms that were not found in sputum by QB. Importantly, KT identified *Pseudomonas* in patients #8 and 9, but gram-negative rods were not seen on Gram stain, and *Pseudomonas* was not detected by QB; these patients responded well to treatment with ceftriaxone and azithromycin.

**Table 1.** Quantitative bacteriologic in sputum vs DNA detection in plasma: patients with clearly established etiology for pneumonia

Pt #	Hours anti-biotics	Sputum WBC/ml	Quantitative bacteriology (cfu/ml sputum)	Karius plasma test (DNA molecules/ul)	Concor-dance	Septic	Lung disease	Immuno-suppressed	Comment
1	2	2.8 × 10^7^	*S. pneumoniae* (1 × 10^7^)	*S. pneumoniae* (60)	Full	Yes	COPD	Yes	Human meta-pneumovirus
2	0	3.2 × 10^6^	*H. influenzae* (6 × 10^6^),	*H. influenzae* (245)	Full	No	COPD	Yes	
3	0	2.6 × 10^7^	*H. influenzae* (2 × 10^8^),	*H. influenzae* (127)	Full	Yes	No	No	
4	0	1.3 × 10^7^	*H. influenzae* (3 × 10^8^),	No organism detected	QB+ Karius−	Yes	COPD	No	Rhinovirus; respiratory failure
5	2	2.2 × 10^8^	*H. influenzae* (2 × 10^6^), *M. catarrhalis* (2 × 10^6^)	No organism detected	QB+ Karius−	Yes	Yes	No	
6	5	2.3 × 10^7^	10^6^ each of *S. anginosus*, *S. salivarius*, *S epidermidis; S. aureus* (1.5 × 10^5^)	*S. pneumoniae* (7,916)	QB− Karius+	Yes	No	No	Bedridden patient, probable aspiration. QB may have missed *S. pneumoniae* because large numbers of other alpha-hemolytic bacteria
7	5	1.8 × 10^7^	Negative for bacteria (<10^3^)	*M. catarrhalis* (81), *S. pneu-moniae* (52)	QB− Karius+	No	No	No	Rhinovirus
8	0	2.4 × 10^7^	*M. catarrhalis* (8 × 10^7^)	*M. catarrhalis* (65), *P. aerugi-nosa* (132)	Partial	Yes	ILD	No	Responded without anti-pseudomonal antibiotics
9	0	6.5 × 10^7^	*S. pneumoniae* (2 × 10^7^)	*S. pneumoniae* (31), *P. aerugi-nosa* (173), *Achromobacter ruhlandi* (108)	Partial	Yes	COPD	No	Responded without anti-pseudomonal antibiotics
10	0	1.8 × 10^7^	*H. influenzae* (1.2 × 10^7^),	*H. influenzae* (773), *S. oralis* (102), *S. sanguinis* (105), *Veilonella parvula* (98)	Partial	No	ILD	No	*M. tuberculosis, M. kansasii* also present; no history of aspiration
11	3	3.2 × 10^7^	*S. pneumoniae* (1 × 10^6^), *S. aureus* (1.2 × 10^8^), *P. aeruginosa* 1 × 10^6^	*S. pneumoniae* (204,000), *S. aureus* (3,821), *P. aeruginosa* (3,007), *N. meningitidis* (943), *Klebsiella* (575)	Partial	Yes	COPD	No	Influenza A, emphysema. Intubated at admission for respiratory failure.

Previous studies of KT have generally been of patients in whom a diagnosis was not established by conventional microbiologic techniques, and the correctness of a positive result was determined by adjudication. In the absence of a microbiologic diagnosis, it is difficult to be certain whether identification of bacterial DNA in plasma represents a true or false positive.^3,4^ In our small cohort of nonbacteremic patients with CAP, clear microbiologic diagnoses were established in every case with discordance between QB and KT in the majority of cases. If the finding, in sputum, of large numbers of typical respiratory pathogens by Gram stain and QB is correct, KT was falsely negative in two cases. The usual cause of discordance (four cases), however, was the finding of bacteria by KT that were not detected by QB. Hogan et al.^5^ showed strikingly similar results with only one-third of patients having fully concordant results; KT regularly identified other organisms that were not found by conventional microbiology. A systematic review^3^ found only 67% positive agreement between conventional microbiology and KT. KT results in our cases might have led clinicians to treat patients #8 and 9 with antipseudomonal antibiotics; they responded to guideline-directed therapy with ceftriaxone and azithromycin.

A positive KT with negative cultures may reflect the presence of noncultivatable bacteria,^6^ but it seems unlikely that, in pneumonia, patients require antibiotic therapy directed against organisms that cannot be seen microscopically or isolated by sputum culture. Detection of molecular material shed from the microbiome^7^ and absorbed into the bloodstream may be responsible for a false-positive KT finding, and such shedding might be increased when acute inflammation is caused, as it occurs in pneumonia.

Detection of bacterial DNA in plasma might be particularly problematic in patients who are immunocompromised because it is very difficult to exclude the possibility of a true positive. A recent study in such patients^8^ found a variety of bacteria by KT when standard of care techniques were negative. While a positive KT could recognize a pathogenic organism, a false positive could lead providers not well versed in the limitations of KT assay to prescribe unnecessary antibiotics, especially in immunocompromised hosts. In summary, discordance between QB and KT in our and other studies suggests that, as attractive as the concept of identifying bacterial DNA in the plasma of infected patients might be, more work is needed to define the clinical usefulness of KT in these infections.
